# Decoding the Dynamics of Circulating Tumor DNA in Liquid Biopsies

**DOI:** 10.3390/cancers16132432

**Published:** 2024-07-01

**Authors:** Khadija Turabi, Kelsey Klute, Prakash Radhakrishnan

**Affiliations:** 1 Eppley Institute for Research in Cancer and Allied Diseases, University of Nebraska Medical Center, Omaha, NE 68198, USA; 2 Fred & Pamela Buffett Cancer Center, University of Nebraska Medical Center, Omaha, NE 68198, USA; 3 Division of Oncology and Hematology, Department of Internal Medicine, University of Nebraska Medical Center, Omaha, NE 68198, USA

**Keywords:** circulating tumor DNA, digital droplet PCR, NGS, minimal residual disease, early detection

## Abstract

**Simple Summary:**

From detection to monitoring therapeutic response, circulating tumor DNA has emerged at the forefront of numerous clinical applications in oncology. It provides a non-invasive, real-time window into the evolving tumor as the cancer progresses. Advances in liquid biopsies and circulating tumor DNA analysis are enhancing their diagnostic applicability with tools like liposomal nanoparticles and antibody priming agents by binding to cell-free DNA in circulation and aiding in evading clearance mechanisms. The development of highly sensitive techniques such as digital droplet PCR and next-generation sequencing-based technologies has enabled the detection of minute ctDNA fractions amid high background, increasing its potential for early detection and minimal residual disease.

**Abstract:**

Circulating tumor DNA (ctDNA), a fragment of tumor DNA found in the bloodstream, has emerged as a revolutionary tool in cancer management. This review delves into the biology of ctDNA, examining release mechanisms, including necrosis, apoptosis, and active secretion, all of which offer information about the state and nature of the tumor. Comprehensive DNA profiling has been enabled by methods such as whole genome sequencing and methylation analysis. The low abundance of the ctDNA fraction makes alternative techniques, such as digital PCR and targeted next-generation exome sequencing, more valuable and accurate for mutation profiling and detection. There are numerous clinical applications for ctDNA analysis, including non-invasive liquid biopsies for minimal residual disease monitoring to detect cancer recurrence, personalized medicine by mutation profiling for targeted therapy identification, early cancer detection, and real-time evaluation of therapeutic response. Integrating ctDNA analysis into routine clinical practice creates promising avenues for successful and personalized cancer care, from diagnosis to treatment and follow-up.

## 1. Introduction

Cancer is a heterogeneous and dynamic disease, and diverse genetic alterations cause its onset and progression. Early diagnosis and accurate assessment of tumor dynamics are pivotal for effective cancer management. In this context, circulating tumor DNA (ctDNA) has emerged as a promising concept. 

ctDNA is a fraction of cell-free DNA (cfDNA) shed by tumor cells into the bloodstream. It carries the genetic and epigenetic alterations specific to the originating tumor, making it an essential source of information for cancer diagnosis and monitoring [1]. The proportion of ctDNA within cfDNA in samples varies according to the tumor burden. In early-stage tumors, it can be less than 1%, while in patients with advanced cancer and a higher tumor burden, it increases to >10% and can even exceed 40% of the total cfDNA [1]. The detection and analysis of ctDNA have rapidly evolved, driven by advances in sequencing technologies and bioinformatics, providing a non-invasive and real-time window into the tumor’s developing landscape [2]. The significance of ctDNA lies in its potential to enable early cancer detection, monitor treatment response, detect residual disease, and predict prognosis, transforming the field of oncology.

The prognosis and detection of cancer are pivotal aspects of cancer management. Early diagnosis and accurate prognosis have mainly been limited over the years by the constraints of traditional diagnostic tools. Although useful, tissue biopsies are invasive and frequently fail to capture the tumor’s genetic heterogeneity [3]. As a result, there has been a growing need for minimally invasive, sensitive, and dynamic tools to improve cancer diagnosis and prognostication.

Liquid biopsy approaches have recently gained momentum, with ctDNA at the forefront. Based on the analysis of ctDNA, circulating tumor cells (CTCs), and other components or markers in the bloodstream, liquid biopsies offer non-invasive and real-time insights into the tumor’s genetic and molecular composition [4]. This paradigm shift has led to multiple advancements in cancer management, enabling early detection, individualized therapy selection, and dynamic monitoring.

This review underscores the potential of ctDNA application in clinical practice, spanning from early detection and tumor profiling to minimal residual disease assessment, treatment selection, and therapeutic monitoring. While acknowledging specific limitations that may hinder its widespread application, the review also delves into current advances that can broaden the utility of ctDNA-based liquid biopsies in oncology. Furthermore, it explores avenues for expanding and optimizing its use, enabling more effective cancer management strategies.

## 2. Biology of ctDNA in Blood

**Figure 1:**
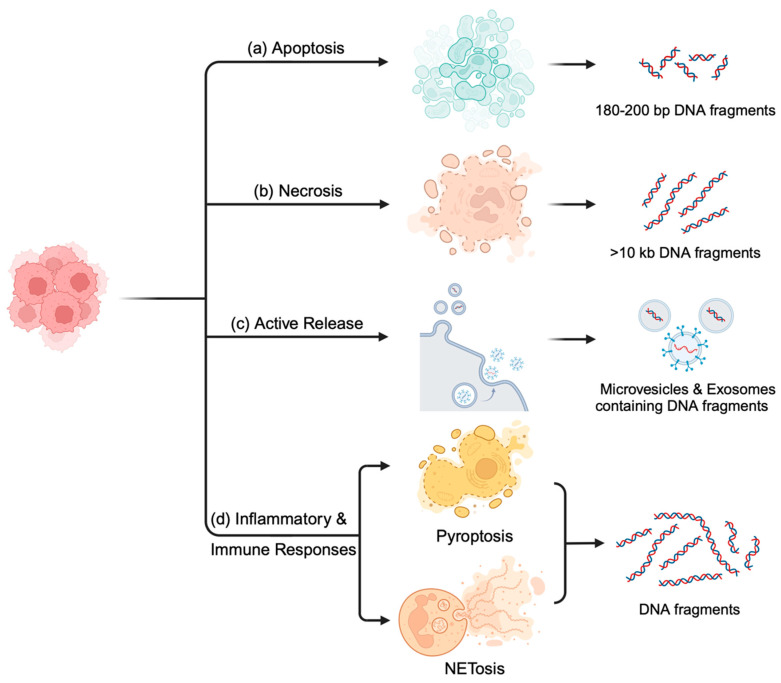
Mechanisms of cfDNA release into the blood circulation. Cell–free DNA, including ctDNA, can be released into the bloodstream as a result of (**a**) apoptosis, which releases short DNA fragments; (**b**) necrosis, yielding longer undigested DNA fragments; (**c**) active release mechanisms such as microvesicles and exosomes; and (**d**) inflammatory and immune responses involving pyroptosis and NETosis.

Distinct mechanisms enabling the movement of DNA from the intracellular to the extracellular milieu, while maintaining its biological stability, are essential to understand. The possible primary origins of cfDNA encompass processes linked to cellular breakdown and active DNA release mechanisms [5]. Methylation studies have shown that hematopoietic cells contribute a major amount of cfDNA in the blood, while only a minor population is associated with other tissues [6]. The average size of cfDNA in healthy individuals, ranging from 160–180 base pairs (bp), reflects its association with nucleosomes (147 bp) and linker DNA (20–50 bp), suggesting a predominant origin from apoptosis [7]. Furthermore, actively metabolizing tissues such as tumors and hematopoietic tissues show increased cfDNA due to increased cell death in such tissues [8]. Even though knowledge on the release mechanisms of ctDNA is quite limited to date, the structural attributes of ctDNA can be traced back to mainly one of these origins, encompassing phenomena such as apoptosis, necrosis, phagocytosis, and active secretion, which underpin specific structural characteristics [9]. Figure 1 enlists the major possible mechanisms associated with cfDNA release.

### 2.1. Apoptosis: Orchestrated Cell-Free DNA Release

The release of cfDNA is heavily associated with apoptosis, a well-organized form of programmed cell death. The primary source of cfDNA within the blood is thought to originate from the hematopoietic cells via apoptosis [10]. During apoptosis, cells undergo a precisely controlled fragmentation process, leading to the generation of relatively short cfDNA fragments, typically around 180–200 base pairs in length [7]. These short fragments are a distinguishing feature, making apoptotic cfDNA easily detectable and analyzable [11]. 

Cancer cells’ distinctive unregulated proliferation results in several stresses, such as paucity of nutrients, inflammatory conditions, hypoxia, oxidative stress, the production and release of tissue-specific transcription factors, and the signaling of death-inducing molecules [12]. Thus, the ctDNA released through apoptosis carries critical information about genomic alterations in cancer cells. It frequently bears specific mutations and epigenetic modifications, making it a valuable tool for cancer diagnosis and monitoring [13]. Furthermore, the induction of apoptosis in cancer cells by drugs can also increase the concentration of ctDNA present in the total cfDNA [14]. In a study evaluating the release of cfDNA in cancer cell lines in response to treatment, it was found that the preliminary cfDNA release associated with treatment was apoptosis, followed by necrosis, and the levels were dependent on the treatment duration and interval [15]. Furthermore, the study also showed that the type of chemotherapy utilized and its targeted cells significantly impacted the cfDNA released. Cytotoxic therapies caused delayed apoptotic cfDNA release in some cells as they underwent senescence, whereas in other cell populations, they led to immediate release as they triggered apoptosis immediately [16]. Therefore, this highlights that cfDNA release kinetics provides essential information for elucidating the therapeutic response and emphasizes the necessity for longitudinal monitoring of ctDNA to maximize its application.

### 2.2. Necrosis: Chaotic but Informative DNA Release

Necrosis represents a less controlled and more chaotic form of cell death compared with apoptosis. It often occurs due to cellular damage or injury, leading to abrupt cell membrane rupturing and the unregulated release of cellular contents, including DNA, into the surrounding environment [17,18]. Necrotic cells exhibit organelle dysfunction and aberrations in the cytoplasmic membrane, causing the tumor DNA to be exposed to the degrading action of intra- and extracellular nucleases and free radicals [12]. Thus, it gives rise to longer and more fragmented ctDNA of more than 10 kb, and the amount of DNA fragments released highly depends on the necrosis-inducing agent [19]. In necrosis, the irregular fragmentation of chromatin leads to the release of DNA fragments of varying sizes, distinguishing it from apoptosis [5]. In solid tumors, where cells at the core can become deprived of nutrients and oxygen, necrosis occurs more commonly [20]. Consequently, the ctDNA profiles can vary, providing information about the tumor size and aggressiveness [21]. 

### 2.3. Active Release: Microvesicles and Exosomes Facilitate Communication

The release of cfDNA is further complicated by active release mechanisms involving microvesicles and exosomes [22]. Microvesicles are released from the cell membrane, which contains various cellular components, including DNA [23]. Furthermore, DNA cargo is transported by exosomes, smaller vesicles originating from multivesicular bodies. These cfDNA-containing vesicles are also released into bodily fluids [24]. Within the context of cancer, ctDNA enclosed within microparticles and exosomes serves as a means of intercellular communication. This communication can influence the tumor microenvironment and potentially contribute to cancer progression and metastasis [8,25]. Exosomes are gaining attention for their role in carrying genetic information between tumor cells and influencing the behavior of both the primary tumor and metastatic sites [26]. Understanding the dynamics of active release mechanisms is essential to comprehending the complexity of cancer progression. These vesicles serve as a means for the surrounding stromal cells and immune cells to interact, which can then dictate tumor behavior.

### 2.4. Inflammation and Immune Responses: Triggers and Modulators of cfDNA Release

Inflammation and immune responses also play a pivotal role in the release of cfDNA, including ctDNA. Inflammatory processes, such as infections, can initiate cell death and the subsequent release of cfDNA [27]. In response to inflammation, immune cells release DNA traps, known as neutrophil extracellular traps (NETs). These DNA traps lead to NETosis, which contributes to the pool of cfDNA in bodily fluids, potentially including ctDNA from tumor cells [19,28]. Furthermore, pyroptosis, an inflammasome-mediated cell death, is another contributor to the cfDNA population [27]. Due to the activation of the inflammasome, the release of pro-inflammatory cytokines leads to cell lysis via pyroptosis. During this process, DNA gets fragmented by nucleases and released into circulation [29]. Tumor-derived cfDNA may carry information about the tumor’s immunogenicity, mutations in immune checkpoint genes, and other factors that influence the response to immunotherapy [30]. 

Another plausible source of ctDNA is the CTCs; however, their contribution to the entire pool of ctDNA is minor. When released from the tumor site, they may undergo breakage release; however, since the number of CTCs is very minimal, their quantification and detection make it challenging to evaluate the mechanism [12]. Despite the various sources and mechanisms of cfDNA and ctDNA release, detecting ctDNA during the early stages of cancer is difficult due to its low abundance within the entire cfDNA population.

## 3. Methods of ctDNA Detection

**Figure 2:**
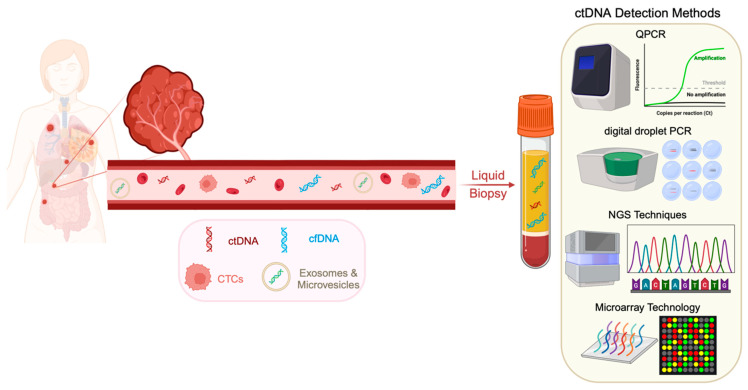
Prevalent ctDNA detection methods utilized for clinical applications. Highly sensitive detection methods facilitate the quantification of low ctDNA fractions. PCR techniques like QPCR, specifically digital droplet PCR, provide high sensitivity and specificity. NGS techniques can also provide targeted and untargeted ctDNA detection approaches. Microarray technology enables concurrently detecting multiple genes and genetic aberrations in ctDNA liquid biopsy samples.

The low amount of ctDNA (as low as 0.01%) poses a significant challenge to its detection. Due to its low concentration, there is a high demand for techniques that can offer sensitivity and specificity for detecting ctDNA among many backgrounds [31]. By tracking a tumor-specific mutation separately from the primary tumor, a directed strategy can track the tumor progression. The methodologies to detect ctDNA can be bifurcated into targeted and untargeted approaches. The most popular targeted approaches include digital PCR (dPCR) and targeted next-generation sequencing (NGS) approaches, or broader, untargeted approaches, including whole genome sequencing (WGS) or global methylation status [32]. Table 1 highlights the advantages and drawbacks of digital PCR-based and NGS-based ctDNA detection methods. The methodology adopted primarily depends on the application of ctDNA analysis, along with the assay sensitivity required to detect varying ctDNA fractions. Figure 2 illustrates the prevalent ctDNA detection methods in liquid biopsies.

The most popular source of ctDNA is from the plasma since the concentration of ctDNA within cfDNA is higher, which improves the detection rate. Furthermore, liquid biopsies are a minimally invasive technique, enabling the enhanced evaluation of the heterogeneity of tumors at multiple time points compared with tissue samples [32]. On the other hand, detecting the signal can be challenging due to the high noise proportion within the plasma samples. 

Techniques such as dPCR have made the detection of point mutations, even in low allele fractions, possible. They enable the detection of rare mutations by analyzing individual target sequences achieved through compartmentalizing complex mixtures [33]. One of the first digital PCR technologies developed was BEAMing (beads, emulsion, amplification, and magnetics), which integrates emulsion PCR and flow cytometry to detect and enumerate variants [34]. Even though it has the ability to detect up to one mutant within 10,000 DNA molecules, the process is quite complex for routine analysis [35]. Digital droplet PCR (ddPCR) is another highly accurate next-generation technique that is based on nanoliter-sized water-in-oil droplet emulsion technology [36]. It can be used for various applications such as rare mutation detection, copy number variation analysis, absolute quantification, gene rearrangements, and DNA methylation from different clinical sample types. ddPCR has a sensitivity ranging from 0.001% to 0.1% and is an effective tool to detect and quantify known point mutations [37]. One significant limitation of ddPCR in ctDNA detection applications is the impact of variability in droplet shape and size on reproducibility and robustness.

Real-time PCR provides a cost-effective and rapid substitute for ctDNA analysis. Even though this method enables minimal false positives, it can effectively detect mutations at a very low frequency of only 10–20% of alleles within wild-type DNA backgrounds, which is considerably low compared with other digital PCR-based methods [31]. Allele-specific PCR (AS-PCR) provides a simple solution for detecting point mutations within ctDNA samples; however, the drawbacks are that it can only evaluate a limited number of foci (as with most targeted approaches) and is only semiquantitative [38]. Co-amplification at lower denaturation temperatures (COLD-PCR) can further detect minute mutation fractions as low as 0.1%. Enriching the mutation fractions can further heighten this sensitivity by 100-fold [39]. The limitations of COLD-PCR are that it only provides a semiquantitative analysis of the mutation load in comparison to some other digital PCR-based techniques and is more prone to polymerase-induced errors [40].

Microarray technology stands out as a powerful tool for ctDNA analysis. Since it is a high-throughput technology, it enables the detection of hundreds or thousands of mutations simultaneously in a single sample [41]. This makes it appealing for multiple applications, particularly when coupled with its affordability and versatility for targeting specific mutations. They also enable the detection of mutations present at low frequencies [42]. The technology operates by immobilizing probes on a solid surface designed to complement specific DNA sequences. Fragmented ctDNA extracted from blood samples is labeled with a fluorescent dye and hybridized into the microarray. The fluorescent signal intensity at each probe location reflects the abundance of the corresponding DNA sequence, allowing the identification and quantification of multiple specific mutations present in the samples [43]. Although these techniques offer many advantages, they are limited in their ability to scan and detect unknown mutations within clinical samples and also exhibit high variance for low-frequency genes.

On the other hand, NGS can profile the ctDNA using both targeted and untargeted approaches and can also be utilized for WGS. Advancements such as unique barcodes or molecular identifiers, which are sequences added to each DNA fragment during library preparation to identify each read or molecule during analysis, help decrease the false-positive rates and enhance the sensitivity of the technology [44]. 

Tagged-Amplicon deep sequencing (TAM-seq) is a method that utilizes NGS, which uses primers to amplify specific genomic regions. Due to its two-stage amplification process, this high-throughput method has a sensitivity of 97% and can detect mutant allele fractions as low as 2% [45]. Furthermore, it has also been developed to determine copy number variants (CNVs), insertions/deletions, and single nucleotide variations (SNVs) [46]. However, the primary limitation of this detection method is its higher detection limit compared with assays targeting individual loci requiring further development, like increased read depth and enhanced algorithms for detection. Another system, known as the Safe-Sequencing System (Safe-SeqS), tags each template with a unique identifier to ensure that a mutation is detected in the majority of the identical unique identifier sequences, providing minimum false positives and improved accuracy and sensitivity [47]. While promising, this sequencing system needs to be validated on larger patient cohorts to accurately evaluate its efficacy. Additionally, further analysis is required to determine the accuracy of the thresholds set for considering positive readouts. Another NGS-based platform is the Ion Torrent, which enables the detection of CNVs and SNVs in amounts as low as 1 ng of DNA [48]. This technology operates by detecting hydrogen ions released during the incorporation of new nucleotides into the growing DNA strand. While it offers faster sequencing, it has a lower throughput compared with some other methods [49].

All the above-mentioned techniques utilize targeted panels that enable the detection of indels and point mutations with high sensitivity and at a lower cost. Alternatively, NGS also provides an untargeted approach that allows WGS to determine CNVs, indels, and point mutations in the entire tumor DNA genome [50]. The drawback of this technique is its reduced sensitivity and increased cost. On the other hand, whole-exome sequencing (WES) provides an improved approach over WGS as it only sequences the exomes within the genome [48]. Nevertheless, the application of these techniques is limited in ctDNA detection and analysis as they require a higher input concentration. Furthermore, it has been found that the sensitivity of digital PCR techniques is higher compared with NGS platforms and can detect smaller mutant fractions [51]. 

An additional useful technique for ctDNA identification in cancer settings is the utilization of DNA methylation markers. In the intricate cancer landscape, characterized by diverse genetic and epigenetic alterations, DNA methylation is one epigenetic change that has been shown to be particularly important [52]. The stability and persistence of DNA methylation patterns make them particularly suitable for ctDNA detection, providing important insights into the tumor’s genetic makeup without requiring invasive procedures [53]. The ability to develop targeted assays is facilitated by the specificity of DNA methylation alterations for specific cancer types, allowing precise diagnostics and contributing to early cancer detection [54]. Moreover, the quantitative nature of methylation analysis enables the evaluation of tumor burden, which helps monitor minimal residual disease (MRD) and assess treatment efficacy [55]. The application of DNA methylation markers in ctDNA detection extends to cancer subtyping, allowing for individualized treatment approaches based on the distinct epigenetic profiles of various cancer types [55]. Methylation-based liquid biopsy tests may soon be able to be entirely integrated into routine clinical practice as challenges related to standardization, sensitivity, and large-scale validation studies continue to be addressed. 

Hence, choosing the most appropriate ctDNA detection method requires various considerations, including the clinical context, desired sensitivity and specificity, target mutations, cost, and availability. NGS offers the most comprehensive analysis but is expensive and requires specialized expertise. Although dPCR has a limited target range, it provides accurate quantification and high sensitivity. Real-time PCR is a cost-effective and rapid option for detecting specific mutations; however, it offers lower sensitivity and specificity. 

### Commercially Available Kits for ctDNA Detection and Analysis

Various kits have been developed for ctDNA analysis from tumor tissues and liquid biopsies due to the increasing attention ctDNA has gained in cancer research over the years. These kits primarily utilize NGS, or PCR-based technology, for analysis. These kits have a variety of applications, including tumor profiling and mutation detection, and some have also been FDA-approved for liquid biopsy tests for various clinical applications in cancer detection and treatment [56,57]. Table 2 enlists some of the commercially available ctDNA kits and their applications.

## 4. Factors Influencing ctDNA Detection

While ctDNA offers invaluable insights into tumor characteristics and dynamics, enabling its application for personalized cancer care, its analysis necessitates careful consideration of certain key factors such as sample collection, preparation, and detection methods. To ensure accuracy and reproducible results, meticulous sample handling and preservation are essential due to the inherent fragility and sensitivity to the degradation of ctDNA.

ctDNA stability is affected by various factors, including the collected sample type, storage conditions, freeze–thaw cycles, and processing time. Plasma ctDNA is generally more stable than serum ctDNA due to degrading enzymes present in the serum [68]. Furthermore, serum samples may contain more background cfDNA than plasma due to the release of necrotic DNA from blood cells [19]. Reducing degradation requires lower temperatures and prompt processing after collection [69]. Additionally, adherence to sterile procedures during blood collection and processing is crucial to prevent contamination with foreign DNA, leading to false-positive results.

Various critical factors influence the successful analysis of ctDNA, including selecting the appropriate collection tubes [69,70]. For instance, a study showed that EDTA tubes are superior to heparin or citrate tubes for ctDNA as the concentration of contaminated DNA is lower in the plasma samples, and EDTA reduces leucocytic apoptosis and necrosis for 24 h [71]. Furthermore, a study found that the stability of cfDNA or ctDNA in EDTA tubes is optimal for up to 6 h [72]. Blood volume depends on the specific analysis and desired ctDNA amount, commonly 10 mL [43]. Tubes containing preservatives offer enhanced stability by preventing degradation, such as Streck Cell-Free DNA BCT^®^ (Streck, USA), Roche Cell-Free DNA Collection Tube (Roche, Switzerland), and Qiagen PAXgene Blood ccfDNA Tube (Qiagen, Germany) [73,74]. Ultimately, understanding the factors affecting ctDNA stability, utilizing proper sample collection and preservation methods, and choosing the correct detection method are key to the accurate and efficient detection of ctDNA. Furthermore, various efforts have been made to manipulate and enrich ctDNA to enhance its detection, as summarized in Table 3 and discussed in the following sections.

## 5. Clinical Applications of ctDNA Analysis in Liquid Biopsies

**Figure 3:**
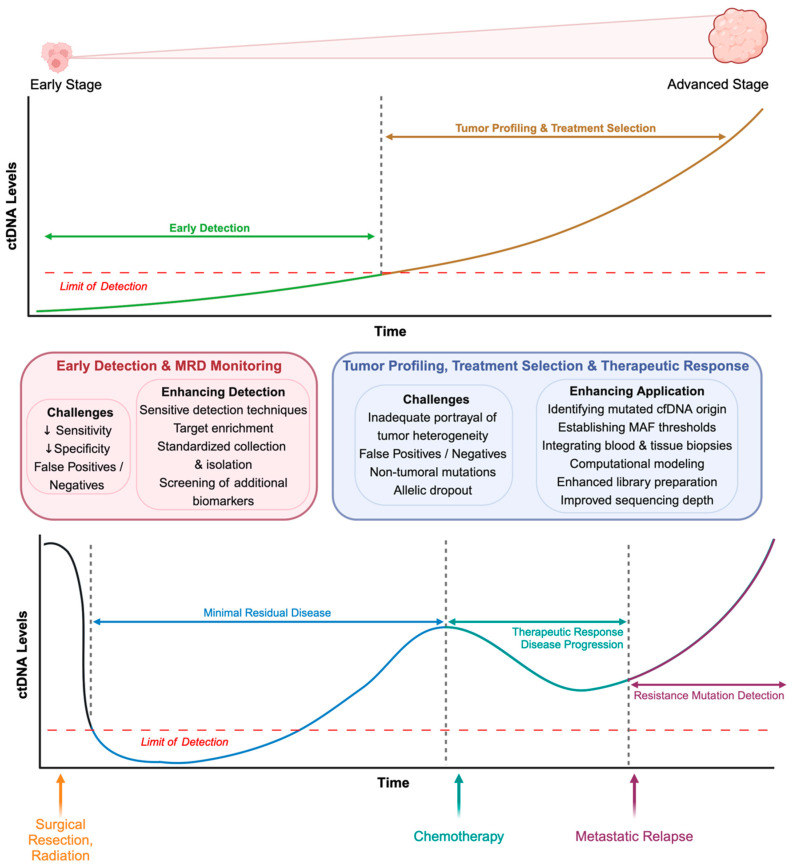
Dynamic levels of ctDNA with cancer progression at different stages for clinical applications. Levels of ctDNA increase proportionally with cancer progression, with almost negligible levels at the early stages of cancer development. Post-intervention ctDNA levels can indicate disease progression, minimal residual disease, and response to treatment. Overcoming the barrier of the limit of detection has been at the forefront of ctDNA research, leading to the development of strategies including target enrichment, priming agents, and computational modelling.

Over the past decade, significant progress has been achieved in understanding the practical applications of ctDNA analysis in several clinical contexts. These include early cancer detection, guiding treatment choices, monitoring minimal residual disease (MRD), and evaluating treatment response [84,85,86]. Figure 3 illustrates the dynamic ctDNA levels and their detection applications at various cancer stages and in response to clinical interventions. ctDNA analysis presents a powerful tool for tailoring therapeutic strategies to the individual requirements of each patient by offering a dynamic overview of the processes involved in cancer progression. This personalized strategy holds immense potential for optimizing treatment efficacy, managing side effects, and improving patient outcomes. Further research and development are necessary to fully realize this potential, but the current momentum suggests that ctDNA analysis can revolutionize cancer care. Table 4 provides an overview of the applications of ctDNA in the analysis of different cancers.

### 5.1. Early Cancer Detection

A highly promising application of ctDNA analysis is in early cancer detection. Traditional methods often involve invasive procedures such as tissue biopsies, which may not be practical for routine screening. A research group developed a specialized capture and sequencing technique known as targeted error correction sequencing (TEC-Seq) to enable the sensitive and specific detection of low-frequency sequence alterations using NGS in cfDNA samples [44]. They employed a 58-gene panel to identify rare tumor-specific alterations in cfDNA samples from patients with various stages of colorectal, lung, breast, and ovarian cancers. TEC-Seq demonstrated the capability to detect ctDNA alterations in 50%, 67%, 45%, and 67% of patients with stage I colorectal, ovarian, lung, and breast cancers, respectively [44]. Although the fraction of patients with ctDNA alterations increased from stages II to IV across all cancers, the data indicated that this methodology could detect ctDNA alterations in stage I of certain cancers [44]. Another study enhanced cancer personalized profiling using the deep sequencing (CAPP-Seq) method for analyzing ctDNA to facilitate lung cancer screening [146]. Despite low levels in early-stage lung cancers, ctDNA is detectable before treatment and strongly prognostic. Most somatic mutations in the cfDNA of lung cancer patients and controls are linked to clonal hematopoiesis [146]. The study develops and validates a machine-learning method, lung cancer likelihood in plasma (Lung-CLiP), demonstrating the potential of cfDNA for lung cancer screening by robustly discriminating between early-stage lung cancer patients and controls [146].

However, the minimal concentration of ctDNA within the entire cfDNA population restricts the potential application of liquid biopsies and ctDNA for early cancer detection. Figure 3 depicts the low levels of ctDNA during the early stages of cancer that go untraced due to the limited detection of current techniques. Despite this challenge, ongoing advancements in molecular techniques are progressively expanding the horizons of liquid biopsy technology, offering hope for broader and more efficient use in early cancer diagnosis.

### 5.2. Treatment Selection and Personalized Medicine

By identifying specific genetic alterations that can guide the selection of targeted therapies, ctDNA analysis plays a pivotal role in guiding treatment decisions. In the age of precision medicine, understanding the genomic landscape of a patient’s tumor is essential for modifying therapies that target the underlying molecular abnormalities driving cancer. Figure 3 shows how evaluating ctDNA at different stages during cancer progression and intervention can help guide treatment selection.

For instance, in patients with metastatic colorectal cancer, ctDNA analysis can reveal mutations in genes such as KRAS and BRAF, which have implications for the response to anti-EGFR therapies [147,148]. Similarly, in advanced gastric cancer, the identification of HER2 amplification through ctDNA analysis guides the use of HER2-targeted therapies like trastuzumab [149]. Another study conducted a phase 2a multicohort trial to evaluate the efficacy of ctDNA monitoring in advanced breast cancer and its ability to guide mutation-directed therapy [103]. Among 1034 patients with ctDNA results, ctDNA testing demonstrated high sensitivity (93%) and specificity (96–99%), allowing for rapid and accurate genotyping [103]. Targeted therapies in cohorts with HER2 and AKT1 mutations showed clinically relevant activity, suggesting the potential for ctDNA testing to guide mutation-directed therapies in routine clinical practice for advanced breast cancer patients.

Furthermore, another study developed highly sensitive and specific mutation-specific ddPCR assays for real-world cancer management, covering 12 genetic aberrations [150]. Applied to 352 plasma samples, the assays accurately reflected cancer progression, and in 20 cases, ctDNA profiling enabled personalized treatment selection based on actionable gene targets, highlighting their potential in routine clinical practice for precise disease monitoring and personalized cancer management [150]. Thus, integrating ctDNA analysis into clinical decision-making can optimize treatment outcomes by ensuring that patients receive therapies that specifically target the genetic alterations driving their cancer, thereby maximizing efficacy while minimizing unnecessary side effects.

### 5.3. Monitoring Minimal Residual Disease

Following initial treatment, MRD indicates the persistence of cancer cells that may not be detectable by conventional imaging or clinical assessments. The persistence of these remnant tumor cells or disease in patients can remain at low, undetectable levels by imaging or physical exam and eventually lead to cancer relapse. Figure 3 highlights the importance of longitudinal monitoring of ctDNA post-initial intervention in detecting and guiding treatment decisions. Studies have shown that therapy after surgery can help eradicate MRD and prevent relapse [151,152]. DNA analysis provides a sensitive and specific method for monitoring MRD, allowing for the early identification of disease recurrence.

A study found that perioperative ctDNA analysis effectively predicted MRD and relapse risk in non-small cell lung cancer (NSCLC) [86]. In the study, postoperative MRD detection strongly predicted disease relapse, surpassing clinicopathologic variables such as TNM staging in predicting recurrence-free survival and demonstrating that adjuvant therapies had differential effects on patients based on MRD status. Similarly, in a study of resectable pancreatic ductal adenocarcinoma (PDAC) patients, a sensitive NGS technology detected preoperative ctDNA in 37.7% of cases, revealing twelve additional oncogenic mutations exclusively in ctDNA [153]. The findings suggest that optimized NGS approaches, including postoperative ctDNA concentration assessment, could enhance MRD evaluation in resectable PDAC, emphasizing the value of parallel analyses of matched tissues and leukocytes for accurately detecting clinically relevant ctDNA.

Another study found that tumor-naive plasma ctDNA analysis is highly sensitive (95%) and specific (100%) for detecting MRD in oligometastatic colorectal cancer (CRC) patients’ post-neoadjuvant chemotherapy [154]. Despite its feasibility, urine-based ctDNA MRD detection showed lower sensitivity (64%), emphasizing the superior performance of plasma ctDNA for guiding personalized treatment in this context [154]. A study focusing on high-risk early-stage hormone receptor-positive breast cancer (HR^+^ BC) found that all patients with positive MRD testing developed distant metastatic recurrence before overt clinical recurrence, suggesting the potential of ctDNA as an early indicator with implications for future interventions in HR^+^ BC patients [155]. The ability to detect residual disease at a molecular level enables timely intervention, potentially improving outcomes by initiating therapeutic interventions before clinical relapse occurs. 

### 5.4. Assessment of Therapeutic Response and Disease Progression

ctDNA analysis is a dynamic tool for tracking the response to therapy and monitoring disease progression in real time. Alterations in ctDNA levels and the emergence of new genetic changes can provide insights into the tumor’s evolving genomic landscape. Monitoring ctDNA in the context of targeted therapies enables clinicians to assess treatment responses and modify therapeutic strategies appropriately. For example, in NSCLC patients receiving tyrosine kinase inhibitors (TKIs) targeting EGFR mutations, changes in ctDNA levels and the emergence of resistance mutations can be indicative of treatment resistance, prompting a switch to alternative therapies [85]. In a study where the ctDNA was monitored serially in progressive metastatic breast cancer, they observed alterations in resistance genes such as ERBB2, TP53, and PIK3CA with disease progression [156].

Additionally, ctDNA analysis has demonstrated its efficacy in predicting treatment responses to immune checkpoint inhibitors in various cancer types [157,158]. Identifying specific genomic features associated with response or resistance to immunotherapy enables selecting patients more likely to benefit from these novel treatment approaches. As suggested in Figure 3, using ctDNA to monitor cancer’s response to chemotherapies can be extremely helpful for tracking progress and making informed decisions in real-time.

## 6. Overcoming Current Limitations with ctDNA Detection

While ctDNA analysis has transformed the field of cancer diagnostics and monitoring by providing a minimally invasive window into tumor genetics, its transformative potential is currently hampered by various challenges that limit its clinical utility. Certain intricacies of ctDNA analysis in cancer, such as technological hurdles and inherent biological factors, impact its applicability and efficacy [2]. Table 3 summarizes the current prominent limitations and the advances made to enhance ctDNA detection.

Achieving high sensitivity and specificity is one of the primary challenges in applying this analysis. ctDNA often appears in minute fractions among a vast background of normal cell-free DNA, making its detection challenging. Low-level mutations or alterations may go undetected, leading to false negatives. Improving the sensitivity of ctDNA assays is crucial for reliable detection, especially in earlier cancer stages where ctDNA levels are relatively low. Liu et al. demonstrated that the enrichment of shorter cfDNA fragments (90–150 bp) by developing an ssDNA library using magnetic beads could significantly improve the sensitivity for low variant allele frequency detection [76]. Even though this study presents a potential solution to enrich ctDNA, it needs further validation, as it is limited by the small patient cohort and the quality and quantity of the samples used for the analysis, which can lead to discrepancies in ctDNA detection.

Additionally, attaining high specificity is essential to avoid false positives, which could result from various factors, including clonal hematopoiesis of indeterminate potential (CHIP) or an increase in non-tumor-derived genetic alterations accumulating in bone marrow and blood cells in a significant fraction of patients [82]. Distinguishing between ctDNA and normal cell-free DNA requires advanced assays, bioinformatic algorithms, and quality control measures [159]. The Signatera™ ctDNA is one such assay that allows for distinguishing tumor-derived genetic alterations from background sources such as CHIP by leveraging ultra-deep NGS and WES data from patients’ buffy coats to eliminate CHIP interference [82]. Factoring CHIP mutations during ctDNA detection can reduce biological background noise by eliminating false positives; however, CHIP mutations can also be indicative of chemotherapy-associated malignancies in cancer patients. Thus, more research needs to be conducted to assess the clinical significance of monitoring CHIP mutations for therapy assessment and disease chemotherapy monitoring [160]. Another technique developed to minimize false positives is AccuScan. AccuScan enhances the potential of MRD by removing errors introduced during DNA sequencing. It integrates linked reads and rolling circle amplification to address errors propagated during library preparation and sequencing [161]. This enables selective amplification of tumor-derived mutations, enhancing the signal and accurately detecting frequencies less than 10 ppm [161]. Even though this technology shows promise, validation in larger patient cohorts in multiple cancers, along with CRC, is essential to determining the applicability of this technology in multiple clinical settings.

**Figure 4:**
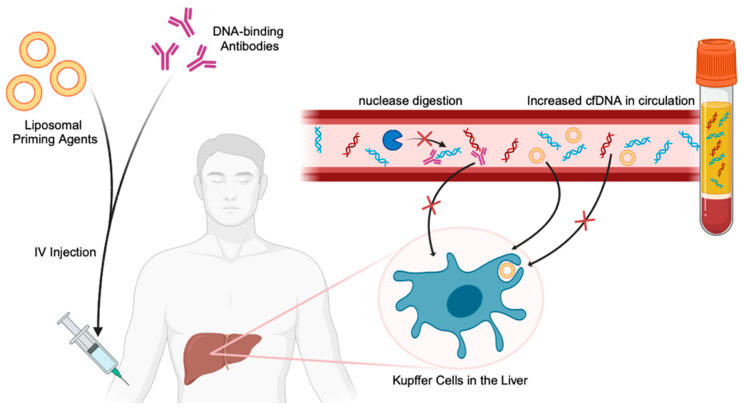
Priming agents to stabilize cfDNA and ctDNA in the bloodstream. Intravenous injections of liposomal nanoparticle priming agents containing succinyl phosphoethanolamine or DNA–binding Fcγreceptor–modified recombinant antibodies inhibit cfDNA (including ctDNA) uptake and degradation by liver macrophages and nucleases, consequently elevating cfDNA concentrations in liquid biopsies.

The stability of cfDNA in circulation is another challenge that limits its implementation. The concentration of ctDNA in the blood is significantly low; furthermore, upon release, it undergoes degradation primarily due to liver-resident macrophages and nucleases in circulation [75]. In a recent study, Martin-Allonso et al. presented two novel priming strategies enabling the elongation of the half-life of cfDNA. A liposomal nanoparticle agent containing succinyl phosphoethanolamine was found to reduce the rate of cfDNA uptake and degradation by the liver macrophage population (kupffer cells) [75]. Another priming agent that showed efficacy was a monoclonal antibody specifically binding to circulating dsDNA, preventing nuclease-mediated degradation, and the engineered structure of the antibody’s Fc domain also enabled evasion of Fc gamma receptor (FcγR)-mediated clearance in the liver [75]. These priming agents, administered 1–2 h before a blood draw, showed the ability to increase the cfDNA up to ten times more than a standard blood draw. The proposed priming agents show great potential for boosting the sensitivity of liquid biopsies; however, further validation and testing of these agents and their required doses is essential to determining how these preclinical findings will translate clinically. Figure 4 summarizes the priming strategies enabling the stabilization and increase of cfDNA in the bloodstream.

Tumor heterogeneity is a significant challenge in ctDNA analysis. Tumors are complex, consisting of diverse cell populations with distinct genetic profiles, and ctDNA may not entirely represent this complexity. Subclonal mutations or alterations in only a small number of tumor cells may be missed, leading to an incomplete understanding of the tumor’s genomic landscape. Comprehending and addressing intra-tumoral heterogeneity is crucial for accurately characterizing the genetic features of the cancer through ctDNA analysis. Two independent studies utilized ctDNA data using NGS and machine learning to determine the comprehensive genomic landscape and biological features defining non-CRC gastrointestinal and metastatic breast cancer [162,163].

Clonal evolution and the emergence of resistance mechanisms are facilitated by dynamic changes in the genomic landscape of tumors during treatment. Even though ctDNA analysis provides real-time information, it might be unable to keep up with how the tumor evolves. Resistance mutations or genetic aberrations may arise, leading to insufficient treatment response assessment and potentially directing clinicians toward ineffective therapeutic strategies. Monitoring clonal evolution and understanding the dynamics of resistance mechanisms present ongoing challenges in ctDNA analysis [164]. To overcome this challenge, a study employed a longitudinal monitoring strategy for ctDNA in metastatic CRC patients, successfully identifying distinct resistance-linked mutations and detecting ctDNA progression before radiological progressive disease, essentially aiding in the identification of potential candidates for clinical trials and targeted therapies [165]. 

The absence of standardized protocols and harmonization across different ctDNA analysis platforms is a significant challenge. Variability in sample processing, sequencing technologies, and bioinformatics pipelines can lead to discrepancies in results between different laboratories or studies [166]. Ensuring the reproducibility and reliability of ctDNA analysis across various clinical settings requires standardizing pre-analytical and analytical procedures and establishing rigorous quality control measures. While the potential benefits of ctDNA analysis are substantial, the associated costs and accessibility remain significant barriers. High-throughput sequencing technologies and complex analytical tools can be expensive, limiting widespread adoption, especially in resource-constrained healthcare settings. For liquid biopsies to reach their maximum potential, efforts to develop cost-effective ctDNA analysis platforms and strategies to improve accessibility are crucial.

Validating ctDNA analysis for clinical application requires stringent clinical trials and regulatory approval. It is a complex process to establish the clinical validity and applicability of ctDNA assays in various cancer stages and types. As ctDNA analysis and liquid biopsies become increasingly integrated into clinical practice, ethical and legal considerations come to the forefront. Issues related to patient consent, data privacy, and the potential psychological impact of ctDNA results on individuals must be carefully addressed. Implementing ethical guidelines is essential for the responsible and patient-centered application of the technology.

## 7. Conclusions

Despite the challenges and limitations, ctDNA analysis can potentially transform cancer diagnostics and monitoring. Ongoing research and technological advancements are gradually addressing many of the existing limitations. Improving sensitivity and specificity, understanding tumor heterogeneity, and standardizing procedures are essential for enhancing the reliability and clinical utility of ctDNA analysis. As the field advances, collaborative efforts are vital to overcoming these challenges. The broader integration of ctDNA analysis into routine clinical practice will be enabled by overcoming technical limitations and enhancing analytical robustness, offering an effective tool for personalized cancer care. Developing this diagnostic tool can have a significant impact on patient outcomes. It allows for closer monitoring of minimal residual disease and the detection of cancer at earlier stages, which often go undetected by most current imaging and diagnostic techniques.

Currently, the most promising technique for ctDNA detection for applications specifically early detection and recurrence monitoring is digital droplet PCR, as it provides a higher sensitivity and specificity for the detection of multiple known mutations. Additionally, incorporating ctDNA stabilization techniques, such as priming agents, and ctDNA enrichment techniques, such as enriching shorter DNA fragments, can further enhance the potential of ddPCR for these cancer detection and monitoring applications. Therefore, despite current obstacles, the momentum surrounding ctDNA research points to a promising future for liquid biopsy as a transformative approach in the cancer diagnostics and monitoring landscape.

## Figures and Tables

**Table 1:** Summary of advantages and disadvantages of digital PCR and NGS-based techniques currently used for ctDNA detection and in various commercial ctDNA kits.

	Digital PCR-Based ctDNA Detection	NGS-Based ctDNA Detection
Advantages	Increased sensitivity (0.1–0.001%)	Enables comprehensive analysis—multiple targets, whole exome, and whole genome.
	Provides absolute quantification of mutation load	Provides an unbiased discovery approach.
Disadvantages	Targeted analysis—detection of known mutants only	Expensive with increased processing times and advanced analysis and data interpretation techniques.
		Higher variant allele frequencies required for detection.

**Table 2:** Application of commercially available kits for ctDNA analysis in cancer.

Name	Technology	Application	Sensitivity	Specificity	Current Clinical Trial ID(s) (Type; Cancer)	Reference
Personalized Cancer Monitoring (PCM™)	Anchored Multiplex PCR (AMP) (for target enrichment) for NGS	MRD	99.8%	99.9%	NCT05219734 (Observational; Pan-cancer)	[58]
TruSight Oncology 500	Targeted NGS	Cancer recurrence detection, Tumor Profiling	>75%	99.9%	NCT05763472 (Observational; Ovarian Cancer) NCT05111067 (Observational; TNBC)	[59]
RaDaR™ (Residual Disease and Recurrence)	Multiplex PCR-based NGS assay	MRD, Early detection of relapse	95%	100%	NCT05388149 (Phase 2; Breast Cancer	[60]
Signatera™	Multiplex-based assay	MRD, Cancer recurrence detection, Therapy Monitoring	>65%	99.9%	NCT04761783 (Observational; Melanoma, NSCLC, CRC) NCT05212779 (Observational; Epithelial Ovarian Cancer) NCT05757843 (Interventional; NSCLC) NCT04786600 (Interventional; CRC) NCT05174169 (Interventional; Colon Cancer)	[61]
MRDetect	WGS	MRD	82–86%	82–93%	-	[62]
Guardant360^®^ CDx	NGS	Therapy Monitoring	55.6%	100%	NCT05935384 (Observational; NSCLC, CRC, Breast Cancer)	[63]
PhasED-seq (Phased Variant Enrichment and Detection Sequencing)	Hybrid capture-based NGS assays	MRD	95%	97%	NCT04417803 (Interventional; Lymphoma	[64]
AVENIO ctDNA Surveillance Kit	CAPP-seq	MRD, Monitoring tumor burden, Therapy Monitoring	95–94%	100%	NCT04585477 (Phase 2; NSCLC) NCT04585490 (Phase 3; NSLC)	[65]
Oncomine Pan-cancer cell-free assay	NGS	Mutation Detection	90%	>98%	NCT04564079 (Observational; NSCLC)	[66]
FoundationOne^®^ Liquid CDx	High throughput hybridization-based capture technology	Mutation Detection	96.3% (PPA) *	99.9% (NPA) **	NCT05272423 (KRAS-driven cancers; Observational) NCT05032092 (Locally Advanced/Metastatic Cancers; Interventional) NCT05846594 (Lung & Gastrointestinal Cancer; Interventional) NCT04484636 (Multiple Cancers; Interventional)	[67]

* PPA: Positive Percent Agreement. ** NPA: Negative Percent Agreement.

**Table 3:** Tools for manipulation and enrichment of ctDNA to improve its application in clinical settings.

Current Challenge	Technology	Application	Reference
Instability of ctDNA and cfDNA	Liposomal nanoparticle priming agents	Inhibits the uptake and degradation of cfDNA (including ctDNA) by liver macrophages and nucleases.	[75]
Background Noise (limiting analytical sensitivity)	Magnetic bead-based isolation, ssDNA library preparation	Enriches shorter ctDNA fragments to enable sensitive detection in low variant allele frequency samples.	[76,77,78]
	Tri-nucleotide Error Reducer (TNER)	Background polishing algorithm that detects and eliminates background mutation errors from healthy subjects and sequencing artifacts from liquid biopsy data.	[79]
	Integrated Digital Error Suppression (iDES)	In silico background polishing to reduce common background artifacts and recover cfDNA molecules by molecular barcoding.	[80]
	INtegration of VAriant Reads (INVAR)	Molecular barcoding and locus-specific background polishing and detection.	[81]
False Positives (non-tumor-derived genetic alterations)	Signatera™ Assay	Filters false positives due to clonal hematopoiesis of indeterminate potential (CHIP).	[82]
	Elimination of Recurrent Artifacts and Stochastic Errors Sequencing (ERASE-seq)	Reduces false positives (10–100 fold) using deconvolution, iterative sequencing of background/negative DNA controls, and technical replicate analysis.	[83]

**Table 4:** ctDNA analysis for various applications in cancer.

Cancer Type	Application	Technology	Total Patients	Reference
Lung Cancer	Therapy Response	NGS	13	[87]
	Therapy Response	NGS	12	[88]
	MRD, Therapy Response	Targeted NGS	139 (97.8% sensitivity)	[89]
	Therapy Selection	Guardant360™ NGS platform	170	[90]
	Therapy Response	Targeted NGS	42	[91]
	MRD, Therapy Response	dPCR	40	[92]
	MRD, Recurrence Monitoring	Multiplex PCR, NGS (RaDaR™ Assay)	88 (86.7% sensitivity; 98.5% specificity)	[46]
	Prognosis	Real Time-Methylation-Specific PCR	42	[54]
	MRD	Targeted NGS	33 (57% sensitivity)	[93]
	MRD, Recurrence Monitoring, Treatment Selection	NGS	330	[86]
	MRD, Therapy Response	CAPP-seq	65	[94]
Breast Cancer	Prognosis, Therapy Response	Targeted capture-based NGS	70	[95]
	Therapy Response	Targeted NGS, SNV detection (Mutect)	88	[96]
	Prognosis	Guardant360™ NGS platform	703	[97]
	Prognosis, Treatment Selection	Hybridization capture & targeted deep sequencing	93	[98]
	MRD, Recurrence Monitoring, Treatment Selection	WGS, Hybrid-capture duplex MRD Test	139	[99]
	Therapy Response, MRD, Metastasis Detection	dPCR	208 (99.8% sensitivity)	[100]
	Therapy Response, MRD, Metastasis Marker	WES, multiplex PCR, NGS	291	[101]
	MRD, Recurrence Monitoring	Hybrid capture-based NGS	142	[102]
	Treatment Selection	ddPCR, Guardant360™ NGS platform	1034 (93% sensitivity; 96–99% specificity)	[103]
	Therapy Response, MRD	TARDIS (Tumor-specific Analysis of Residual Disease in Solid Tumors)	33 (19.6–94.6% sensitivity; 100% specificity)	[104]
Pancreatic Cancer	Therapy Response, Treatment Selection	ddPCR	69	[105]
	Prognosis	Guardant360™ NGS platform	44	[106]
	Prognosis	ddPCR	55	[107]
	Metastasis Marker	ddPCR	172	[108]
	Therapy Response	dPCR	47	[109]
	Therapy Response	Oncomine Colon cfDNA Assay targeted NGS	106	[110]
	Prognosis	NGS	112	[111]
	Mutation Detection	ddPCR	162	[112]
	Prognosis, Therapy Response	ddPCR	67 (0.01–0.1% sensitivity)	[113]
	Therapy Response	Targeted NGS	38	[114]
	Prognosis, Therapy Response	NGS, ddPCR	188	[115]
Colorectal Cancer	Cancer Detection	Methylation-specific PCR	212 (43.1% sensitivity; >85.9% specificity)	[116]
	Cancer Detection	Targeted Methylation Assay by NGS	20 (85% sensitivity; 92% specificity)	[117]
	MRD	Multiplex QPCR	299 (78% sensitivity; 90.2% specificity)	[118]
	Mutation Profiling	Targeted NGS	23	[119]
	Therapy Monitoring	WES, Targeted NGS	171	[120]
	Therapy Selection	ddPCR	33 (80% sensitivity; 90% specificity)	[121]
	Prognosis	ddPCR	48 (93% sensitivity; 95% specificity)	[122]
	Therapy Response	ddPCR, NGS	28	[123]
	Therapy Selection	ddPCR	100	[124]
	Cancer Detection, Mutation Detection	ddPCR	155 (45% sensitivity; 100% specificity)	[125]
Skin Cancer	Therapy Response	AS-PCR, RT-PCR, ddPCR	85	[126]
	Disease Progression	ddPCR	93	[127]
	Recurrence Monitoring	ddPCR	133	[128]
	MRD	Signatera™	69	[129]
	Prognosis	ddPCR	174	[130]
	Prognosis	ddPCR	80	[131]
	Therapy Response	ddPCR	72	[132]
	Therapy Response	ddPCR	96	[133]
Prostate Cancer	Disease Progression	WGS; TAM-Seq	10; 189	[134]
	Mutation Profiling, Therapy Response	Guardant360™ NGS platform	514	[135]
	Mutation Profiling	Hybrid capture–based comprehensive genomic profiling	3334	[136]
	Mutation Detection	FoundationOne^®^ NGS	619	[137]
	Mutation Detection, Mutation Profiling	NGS	279	[138]
	Mutation Profiling	Targeted NGS sequencing	100	[139]
	Prognosis	Targeted NGS sequencing	491	[140]
Head & Neck Cancer	Mutation Profiling	Guardant360™ NGS platform	60	[141]
	Disease Progression	Signatera™	116	[142]
	Mutation Detection	ddPCR	107	[143]
	Mutation Detection	Safe-Seqs	62	[144]
Breast, Liver, Lung, Colorectal & Gastric Cancer	Early Detection, Localization	SPOT-MAS (tumor methylation screening), NGS	738 (73.9–88.3% sensitivity; 97% specificity)	[145]

## Data Availability

Not applicable.

## Abbreviations

AS-PCR	Allele-specific PCR
BEAM	Beads, emulsion, amplification, and magnetics
CAPP-Seq	Cancer personalized profiling by deep sequencing
cfDNA	Cell-free DNA
CHIP	Clonal hematopoiesis of indeterminate potential
CNVs	Copy number variants
COLD-PCR	Co-amplification at lower denaturation temperature-PCR
CRC	Colorectal cancer
CTC	Circulating tumor cell
ctDNA	Circulating tumor DNA
ddPCR	Digital droplet PCR
dPCR	Digital PCR
HR+BC	Hormone receptor-positive breast cancer
Lung-CLip	Lung cancer likelihood in plasma
MRD	Minimal residual disease
NET	Neutrophil extracellular trap
NGS	Next generation sequencing
NSCLC	Non-small cell lung cancer
PDAC	Pancreatic ductal adenocarcinoma
Safe-SeqS	Safe-Sequencing System
SNVs	Single nucleotide variations
TAM-Seq	Tagged-Amplicon deep sequencing
TEC-Seq	Targeted error correction sequencing
TKIs	Tyrosine Kinase Inhibitors
WES	Whole exome sequencing
WGS	Whole genome sequencing
